# IgE-expressing long-lived plasma cells in persistent sensitization

**DOI:** 10.3389/fped.2022.979012

**Published:** 2022-12-05

**Authors:** Shiqiu Xiong, Yang Jia, Chuanhe Liu

**Affiliations:** ^1^ Department of Allergy, Center for Asthma Prevention and Lung Function Laboratory, Children's Hospital of Capital Institute of Pediatrics, Beijing, China; ^2^ Department of Pediatrics, Graduate School of Peking Union Medical College, Beijing, China; ^3^ Department of Pediatrics, The Second Xiangya Hospital, Central South University, Changsha, China

**Keywords:** long-lived plasma cells, IgE, B cell receptor, microenviroment, class switch recombination, persistent sensitization

## Abstract

Persistent allergies affect the quality of life of patients and increase economic burdens. Many clinical observations indicate the presence of IgE^+^ long-lived plasma cells (LLPCs), which account for the persistent secretion of specific IgE; however, the characteristics of the IgE^+^ LLPCs have yet to be identified clearly. In this review, we summarized the generation of IgE^+^ PCs, discussed the prosurvival factors in the microenvironment, and reviewed the unique IgE-BCR signaling, which may bring insights into understanding the survival mechanisms of IgE^+^ LLPCs.

## Introduction

Allergic diseases are a global health problem and a significant burden to society and patients. Over the past two decades, the prevalence of various allergic diseases has increased (1, 2). Allergic sensitization during childhood is a dynamic process. Some allergies can outgrow naturally, especially food allergies, such as cow milk allergy, egg allergy, and wheat allergy (3). According to a study by Bernard and colleagues, the remission rate of aeroallergen allergy within 2 years was 60% (4). However, some allergies (e.g., tree nuts, peanuts, fish, shellfish) are lifelong (3). People with persistent sensitization have an increased risk of developing allergic diseases, such as asthma and allergic rhinitis, thus impairing patient quality of life and incurring substantial economic costs (5, 6). Up to date, allergen immunotherapy (AIT) is the only way to cure allergies. AIT for dust-mite, pollen, milk, egg and peanut allergies has been used widely. However, the shortcomings, such as the long duration of therapy, potential adverse reactions, unclear long-term effects, and limited applications, make it challenging to meet clinical requirements (7). Thus, it is essential to understand the mechanism of persistent allergy and develop rational therapies to shorten the duration of allergy.

Long-lived plasma cells (LLPCs) are critical for maintaining adaptive humoral immunity after recovery from infection or vaccination. However, LLPCs also generate pathogenic antibodies, thus causing a variety of diseases or problems, such as systemic lupus erythematosus, allograft rejection, and persistent allergy (8, 9). IgE is the critical factor of type I hypersensitivity and mediates the degranulation of mast cells and basophils, leading to the rapid manifestation of symptoms after allergen exposure. The half-life of serum IgE is short [2–3 days in humans (10) and 12 h in mice (11)]. In contrast, allergen-specific IgE could maintain for an extended period with the absence or significantly attenuated exposure of allergen both in mice (12) and humans (13, 14), which suggests that IgE^+^ LLPCs may account for persistent IgE secretion (15). To clarify the potential role of IgE^+^ LLPCs in allergic conditions, we summarized the generation of IgE^+^ plasma cells (PCs), discussed the prosurvival factors in the microenvironment, and reviewed the IgE-BCR downstream pathways which uniquely regulate IgE^+^ PCs longevity.

## Evidence of IgE^+^ LLPCs in mice and humans

Much evidence has been raised that IgE^+^ LLPCs were inducible in allergic mouse models. Mice injected intraperitoneally with a single dose of OVA with or without aluminum hydroxide (HA) had a persistent titer of OVA-specific IgE in serum and long-lived IgE-secreting cells in the bone marrow (BM) and spleen. Moreover, administration with X-irradiation, a lethal dose of x-rays sufficient to deplete B memory cells rather than LLPCs, only partially affected IgE levels and IgE^+^ secreting cell counts (12). Another study induced systemic sensitization in mice by intraperitoneal injection with OVA-HA on days 1, 14, and 21 and subsequently treated them with cyclophosphamide for 4 days (16). Researchers found that OVA-specific IgE-secreting cells survived in the BM and lesser in the spleen at day 100. Unlike short-lived plasma cells (SLPCs), LLPCs are refractory to cyclophosphamide, and these IgE-secreting cells were thought to be IgE^+^ LLPCs (16). However, intraperitoneal injection with allergens is not a natural route to allergen exposure. Asrat et al. (17) developed an allergic model by intranasal exposure to house dust mite (HDM) extract. In mice that were chronically exposed to HDM and left unmanipulated for additional periods, the serum IgE decreased initially but maintained constant after 14 weeks. Parallelly, IgE^+^ BMPCs were present in BM for at least 32 weeks and were not significantly affected by anti-CD20 antibodies administration. Because the lifespan of IgE and IgE^+^ SLPCs is short and the absence of allergen exposure and anti-CD20 antibodies administration block the *de novo* IgE^+^ PCs generation, the IgE^+^ PCs persistent in BM were LLPCs and contributed to long-term positive IgE (17). In addition to the aeroallergen allergy, the food allergy mouse model induced by peanut butter with cholera toxin intragastrically also demonstrated that cyclophosphamide-resistant IgE^+^ PCs could be induced and maintained in BM by intestinal allergic sensitization (18). Despite the differences between allergens, routes of allergen exposure, and the genetic background of mice, IgE^+^ LLPCs could be induced and mainly reside in BM.

In humans, many clinical observations indicate the presence of IgE^+^ LLPCs; however, direct evidence is lacking. For instance, with the mugwort pollen dropped, the allergen-specific T cells in patients with mugwort pollen allergy almost disappeared, but allergen-specific IgE persisted for several years (13). In cat-sensitization individuals, the level of specific IgE to cat did not significantly change after avoiding cat allergen for 20 months (14). More recently, Pitlick and Pongdee conducted a study in which patients received combining biologics targeting IgE^+^ IL-5/IL5R, IgE^+^ IL4/IL13, IgE^+^ IL-15^+^ IL4/IL13, and IL-15^+^ IL4/IL13 respectively. During the treatment, IgE levels in some patients cannot decrease to the normal range (19). IgE^+^ BMPCs could be found in allergic patients but not in nonallergic participants, and these IgE^+^ BMPCs secret allergen-specific IgE which could stimulate allergic reactions (17). However, it is difficult to identify the longevity of IgE^+^ BMPCs in atopic individuals. According to Zhang's study, IgE^+^ PCs were found in human nasal polyps and could secrete IgE constantly for 1-month *ex vivo* without stimulation (20). Moreover, a proportion of PCs (BCL2^+^ CD138^+^ PCs) could survive *ex vivo* for at least 32 days, a lifespan of LLPCs in the human intestine, indicating the existence of LLPCs in nasal polyps (20). Nevertheless, this study did not raise direct evidence that IgE^+^ PCs were LLPCs. LLPCs have several features which could distinguish them from SLPCs and B cells. LLPCs are long-lived, cyclophosphamide-resistant (16), and radioresistant (12). At the molecular level, LLPCs express anti-apoptosis proteins (such as MCL1 and BCL2), CD28, and the BCMA receptor (21, 22). These features could help to identify the long-lived population of IgE^+^ PCs in humans.

## Generation of IgE^+^ PCs

### The germinal center (GC)-dependent pathway

Typically, B cells migrate to the GC, a primary place for class switch recombination (CSR) and affinity maturation, after interacting with antigen-dependent T cells. GC B cells further differentiate into PCs under the interaction with follicular dendritic cells and T follicular (Tfh) cells (23, 24). Tfh cells are essential for affinity maturation and class switching of GC B cells. Different profiles of cytokines derived from Tfh cells induce B cells to switch into different isoforms of immunoglobulin (Ig) (24). For IgE-inducing in mice, Tfh cells that secret IL-4 are sufficient for IgE production, typically low-affinity IgE (25, 26). Tfh-derived IL-21 plays a negative role in IgE CSR, which could be attenuated by CD40 signaling (27). A recently identified IL-4^+^ IL-13^+^ Tfh13 population in mice and humans, a subtype of Tfh induced by various allergens, might play a crucial role in high-affinity IgE generation (26). IgE^+^ cells experiencing indirect isotype switching mainly from IgG1 (IgM → IgG1 → IgE) are more likely to produce high-affinity IgE. In contrast, low-affinity IgE derives from IgE^+^ cells undergoing direct class switching (IgM → IgE) (28–30). In humans, IgE can also switch from other Ig isotypes (28, 31).

He et al. analyzed Sγ1 remnants in switch regions of IgE^+^ GC B cells and IgE^+^ PCs in mice immunized with OVA + PEP1 or infected with *N. brasiliensis*. They found that Sγ1 remnants presented in a proportion of IgE^+^ PCs but not in IgE^+^ GC B cells, indicating that IgG1^+^ cells could be the precursors of IgE^+^ PCs besides IgE^+^ GC B cells (32). IgE^+^ GC B cells are transient in GC and are predisposed to differentiate into SLPCs (33). Therefore, IgE^+^ GC B cells are more likely to experience direct class switching and are the precursors of low-affinity IgE^+^ SLPCs. IgG1^+^ PCs are terminally differentiated, so they cannot further differentiate into IgE^+^ PCs (32). IgG1^+^ memory cells and IgG1^+^ GC cells may be the primary IgG1^+^ cells to switch to IgE^+^ PCs, which has been demonstrated by Talay (34, 35) and He (32). IgE^+^ SLPCs also could arise from IgE^+^ memory B cells in mice after the second challenge with *N. brasiliensis* (34, 35). These results are consistent with research based on humans. IgE^+^ memory B cells fitted with the GC-dependent and GC-independent pathways were detected in human peripheral blood samples by analyzing the replication history and SHM levels. *In vitro*, the two types of memory B cells could differentiate into IgE^+^ PCs cultured with anti-CD40 and IL-21 (36).

**Figure 1:**
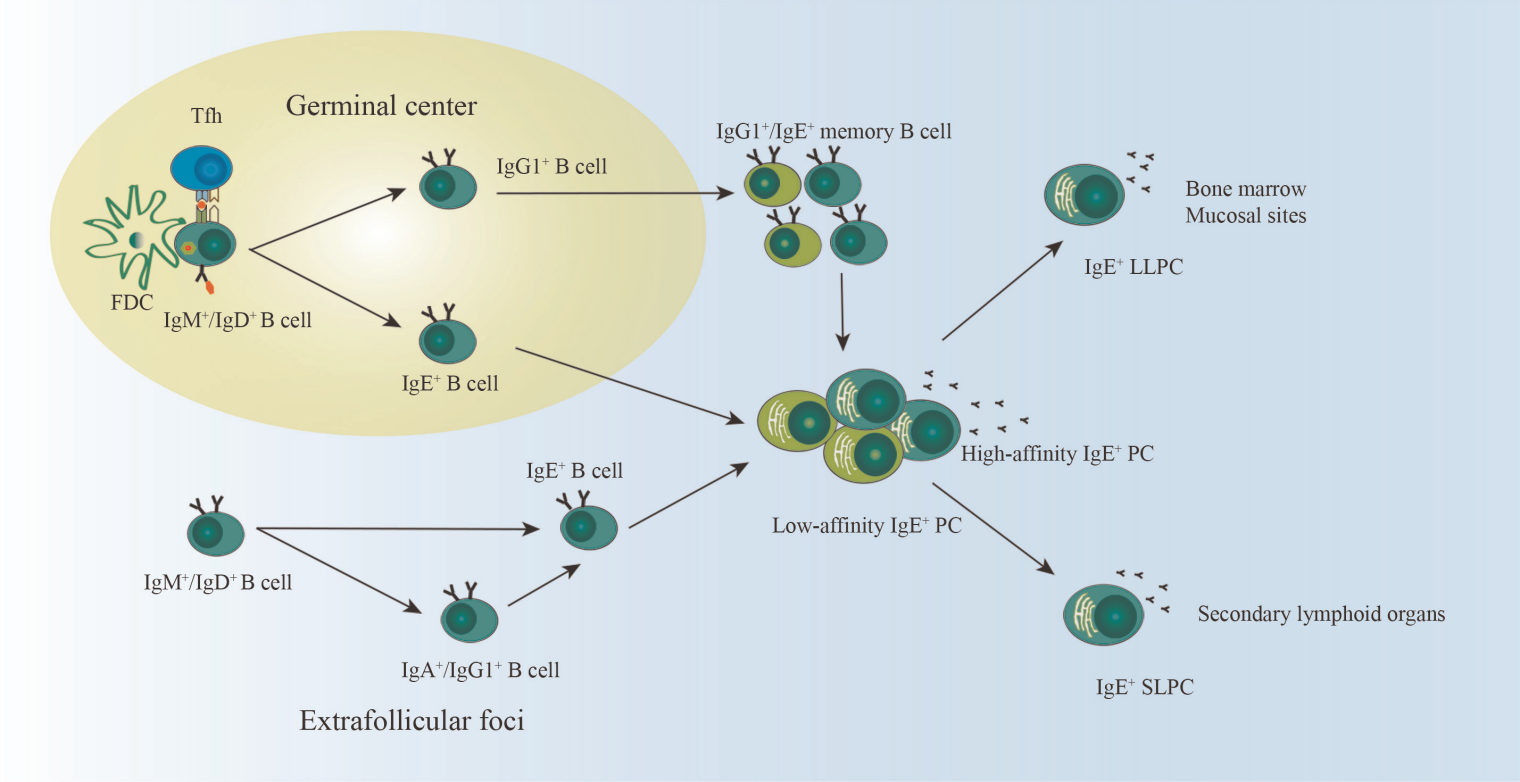
The generation of IgE^+^ LLPCs. With the help of FDC and Tfh cells in the germinal center, most naïve B cells experience somatic hypermutation and class switch recombination, including direct class switching (IgM/IgD → IgE) and indirect class switching (IgM/IgD → IgG1 → IgE). B cells experiencing direct class switching are more likely to differentiate into low-affinity IgE^+^ PCs. High-affinity IgE^+^ PCs may derive from B cells undergoing indirect class switching. IgE^+^ GC B cells, IgE^+^ memory B cells, and IgG1^+^ memory B cells are precursors of IgE^+^ PCs. A small proportion of IgE^+^ PCs migrate into the bone marrow and become LLPCs. SLPCs reside in secondary lymphoid organs. IgE^+^ PCs can also be generated in the extrafollicular foci and are more likely the SLPCs. FDC, follicular dendritic cell; Tfh, follicular helper T cell; PCs, plasma cells; LLPCs, long-lived plasma cells; SLPCs, short-lived plasma cells.

A small proportion of PCs migrate to BM and survive as LLPCs (37). IgE^+^ BMPCs are detectable in mice with *N. brasiliensis* infection, and most of them experience sequential class switching from IgG1 (32). Compared with irradiated mice receiving IgE^−^ PCs, mice receiving IgE^+^ PCs purified from mice immunized with OVA + HA/Alum had higher levels of long-lasting serum IgE as well as increased expression of IgE transcripts in BM and spleen (32). This finding indicates that a proportion of IgE^+^ PCs could migrate to BM and might be the LLPCs. However, direct evidence is lacking (Figure 1).

### The GC-independent pathway

Recently, increasing evidence has shown that IgE^+^ PCs can be derived by the extrafollicular pathway (Figure 1). Kwon1 and colleagues showed that thymic B cells differentiated into IgE^+^ PCs in the thymus and produced natural IgE in mice (38). Another study showed that IgD^+^ B cells could differentiate into IgE^+^ PCs directly (IgD → IgE) or sequentially (IgD → IgA/IgG → IgE) in the nasal mucosa (39). The GC is critical for persistent humoral IgE responses because abnormal GC formation leads to a faster decrease in specific IgE in the peanut allergy mouse model (18). Thus, extrafollicular-derived IgE^+^ PCs are more likely to be SLPCs. GC is the primary place for CSR and affinity maturation. From this viewpoint, impaired GC formation may lead to the generation of low-affinity IgE, which competes with high-affinity IgE and prevents anaphylaxis (29, 40). However, a study by Jiménez-Saiz found that IgE in Bcl-6^B cell^ knock-out mice with impaired GC formation could trigger anaphylaxis (18). Researchers did not further analyze the switch region in DNA fragments of IgE^+^ PCs and did not test the affinity of IgE derived from Bcl-6^B cell^ knock-out mice.

## Mechanisms for IgE^+^ PCs survival

IgE^+^ B cells and IgE^+^ PCs are poorly characterized because of their scarcity and low expression of membrane IgE. We reviewed the microenvironment of IgE^+^ LLPCs and the unique mechanisms mediated by IgE-BCR, which may bring insights to understand the survival mechanisms of IgE^+^ PCs.

### The microenvironment for IgE^+^ PCs survival

LLPCs expressing the chemokine receptor CXCR4 will migrate to the BM, where these cells receive various survival signals. In contrast, SLPCs mostly die within 1 week in secondary lymphoid organs (17, 41). In mice induced chronically by HDM, IgE^+^ BMPCs express CXCR4 at a similar level with IgG1^+^ BMPCs (17). Moreover, IgE^+^ LLPCs induced in mice mainly reside in BM, suggesting that, like other LLPCs, BM is also the primary harbor of IgE^+^ LLPCs (12, 16–18). Additional reservoirs of IgE^+^ LLPCs besides BM were also reported, such as lungs and nasal polyps, implying that prosurvival factors in these mucosal sites contribute to the longevity of PCs (17, 20). The BM microenvironment is essential for PC survival and has been well-reviewed (23). Briefly, the adhesion molecules VAL-4 and LFA-1 are responsible for LLPC retention by interacting with ligands expressed by stromal cells, such as ICAM-1, ACAM-1, and fibronectin (42, 43). In the BM niche, stromal cells and hematopoietic cells (e.g., monocytes, eosinophils, basophils, BM dendritic cells, and megakaryocytes) could offer survival signals through cell-to-cell contact and cytokines, such as a proliferation-inducing ligand (APRIL), B-cell activating factor of the TNF family (BAFF), and interleukin-6 (IL-6) (37).

Identifying the prosurvival factors in the microenvironment other than BM is interesting. Many studies explored the cytology and cytokine profiles in nasal polyps and lungs. A higher level of eosinophils and IL-6 in eosinophilic nasal polyps and bronchoalveolar lavage fluid (BALF) of asthmatic patients was detected (44, 45). These two factors could promote the survival of LLPCs in the BM (43). Increased expression of other cytokines, such as IL-4, IL-13, IL-5, IL-8, et al., was also reported (45–47). IL-4 and IL-13 contribute to the class switching of IgE and promote memory B cells to differentiate into IgE^+^ PCs; however, they may not contribute to survival (48). IL-5 play a role in supporting the survival of BMPC (49). The expression of IL-5R was upregulated in PCs in nasal polyps from subjects with aspirin-exacerbated respiratory disease, and stimulation with IL-5 *in vitro* led to high expression of transcripts related to cell proliferation (50). Multiple myeloma (MM) cells are malignant PCs that share some common characteristics with LLPCs. IL-8 could protect MM cells from cell death induced by serum starvation (51). Collectively, some cytokines and cells that could support PC survival are also present in nasal polyps and lungs. However, the prosurvival role was identified in the BM or *in vitro*; whether these factors play the same effect in nasal polyps and lungs remains to be clarified.

### The IgE-BCR signaling in IgE^+^ PCs

The serum level of IgE (50–200 ng/ml) is the lowest of all the Ig subclasses in non-atopic persons (52). In addition, IgE^+^ cells are rare, and IgE^+^ B cells are likely to differentiate into SLPCs and undergo apoptosis (53). Thus, the IgE production and IgE-secreting cells' lifespan are strictly regulated, or uncontrolled IgE response could cause allergic reactions. Unlike other PCs, IgE^+^ PCs have several unique features. Firstly, IgE^+^ PCs are scarce and short-lived in healthy conditions (33). Besides, IgE-BCR is paradoxically upregulated in IgE^+^ PCs (54). Moreover, IgE-BCR could activate autonomously without antigen engagement (55). Thus, IgE-BCR signaling may play an essential role in IgE^+^ PC regulation, and identifying the specific molecular mechanism helps discover the potential pathogenesis of the allergic disease.

#### IgE-BCR in humans and mice

Membrane IgE (mIgE) has two isoforms sharing the same mRNA precursor but alternative splicing sites. The long form of mIgE (mIgE_L_) has an additional extra-membrane proximal domain (EMPD) region between Cε4 and the transmembrane M1 domain. EMPD contains 52 amino acid residues and exists in some primate species, including humans (56). Other mammal animals, including mice, only express short mIgE (mIgE_S_). In the human B cell line, the amount of mIgE_L_ was much lower than mIgE_S_ on the cell surface due to EMPD. EMPD could act as an autonomous endoplasmic reticulum (ER) retention domain and might restrict the transport of mIgE_L_ from ER to the cellular membrane (56). Moreover, modulation of the two isoforms exists. In human tonsil B cells cultured with anti-CD40 and IL-4, mIgE_L_ was downregulated in IgE^+^ PCs along the differentiation pathway and compensated by mIgE_S_ (54). Humans have the propensity to develop allergic diseases but apparently not in mice, indicating a unique role of mIgE_L_ isoform in allergy development.

Compared to wild-type mice, mice with IgE-BCR mutation show a reduction of IgE^+^ PC population after infection with *N. brasiliensis*, indicating a crucial role of IgE-BCR for PC accumulation (57). *In vitro*, spleen B cells isolated from naïve mice with IgE-BCR (mIgE_S_) mutation and cultured with IL-4 and anti-CD40 antibodies show an impaired formation of IgE^+^ PCs (57). Another study developed murine spleen B cells that only expressed one isotype of BCRs and culture them with IL-4 on feeder cells expressing CD40l and BAFF. Among these isotypes (IgM/IgD/IgA/IgG/IgE-BCR), only IgE-BCR could induce B cells to differentiate into PCs and apoptosis (55). Thus, IgE-BCR could facilitate IgE^+^ PC differentiation and apoptosis in an antigen-independent way. In a human B cell line, stimulating mIgE_S_ of DG75 B cells with anti-IgE antibodies also leads to apoptosis (56). Studies on mIgE_L_ are controversial. Haniuda et al. found that mIgE_L_ could stimulate activation-induced cell apoptosis *in vitro* but is less efficient than mIgE_S_ (56). In contrast, one study developed transfected murine B cells that express immunoreceptors with or without EMPD. A higher proportion of B cells lacking EMPD underwent apoptosis, suggesting a role of EMPD in controlling apoptosis (58). However, the degree to which these findings *in vitro* apply to IgE^+^ PC cells *in vivo* is unclear.

#### The signaling pathways of IgE-BCR

**Figure 2:**
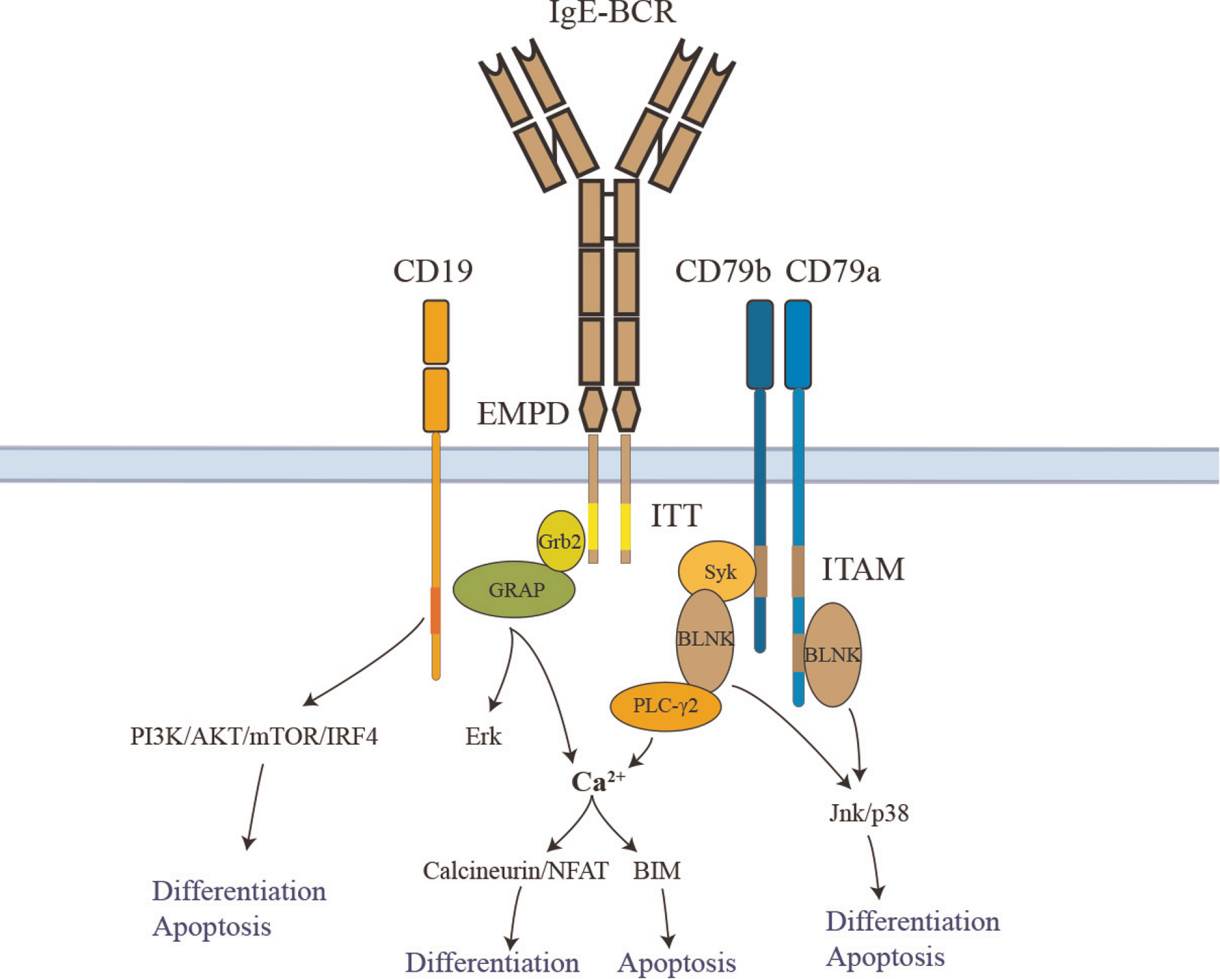
The signaling pathways of IgE-BCR. IgE-BCR is non-covalently associated with CD79a and CD79b, both of which have an ITAM motif in their cytoplasmic tail. Phosphorylated ITAM recruits and activates Syk, which phosphorylates BLNK after activation. In addition, CD79a could recruit and activate BLNK by a non-ITAM tyrosine residue. BLNK controls calcium signaling pathways *via* activating PLC-γ2. The downstream signaling calcineurin/NFAT promotes IgE^+^ PC differentiation. Sustained elevation of calcium contributes to BIM-dependent apoptosis. BLNK also facilitates IgE^+^ PC differentiation and apoptosis *via* Jnk/p38. In addition, the ITT motif recruits Grb2 and Grb2-related adaptor proteins to enhance BCR downstream signaling. The long isoform of IgE-BCR has an additional EMPD region that could interact and activate CD19. Activated CD19 triggers IgE^+^ PC differentiation and apoptosis *via* the PI3K-Akt-mTOR-IRF4 pathway. ITAM, immunoreceptor tyrosine-based activation motif; EMPD, extra-membrane proximal domain; ITT, immunoglobulin tail tyrosine; GRAP, Grb2-related adaptor protein.

Like other BCRs, IgE-BCR is non-covalently associated with CD79a and CD79b signaling devices. However, IgE-BCR could activate the downstream signaling autonomously (59). CD79a and CD79b contain an immunoreceptor tyrosine-based activation motif (ITAM). Phosphorylated ITAM recruits and activates Syk, which phosphorylates BLNK after activation. In addition, CD79a could recruit and activate BLNK by a non-ITAM tyrosine residue. BLNK controls calcium signaling pathways *via* activating PLC-γ2 (60). In contrast to IgM and IgD-BCR, IgE-BCR and IgG-BCR have a cytoplasmic Ig tail tyrosine (ITT) motif, enhancing ITAM-induced calcium signaling as well as Erk MAP kinase pathways (60–62). The IgG-BCR ITT motif could incorporate Grb2/Btk signaling module and amplify downstream signaling. However, the IgE-BCR ITT motif recruits Grb2 and Grb2-related adaptor proteins to enhance BCR signaling (62) (Figure 2).

Haniuda et al. (55) transduced GC-like B cells with mIgE or mIgG1 and examined the activation of the BCR signaling cascades without BCR stimulation. Compared to mIgG1, mIgE showed spontaneous activation of BCR signaling (BLNK-Jnk/p38) and CD19 downstream signaling (CD19-PI3K-Akt-IRF4). *In vitro*, the CD19 pathway was proven to play a crucial role in IgE^+^ SLPCs differentiation but not apoptosis. Further, researchers immunized mice with the TD Ag NP-CGG in alum to clarify the function of CD19 *in vivo*. CD19^+/−^ mice had attenuated IgE^+^ PC differentiation but a long-lasting serum IgE titer. BLNK-Jnk/p38 rather than Erk pathway donated to IgE^+^ SLPCs differentiation and apoptosis. Immunized BLNK^−/−^ mice also had a long-lasting serum IgE titer and generation of IgE^+^ LLPCs (55). Therefore, IgE-BCR triggers SLPCs generation and apoptosis *via* activating BLNK-Jnk/p38 and CD19-PI3K-Akt-IRF4 pathways (Figure 2). The PI3K-mTOR-IRF4 pathway also drives the differentiation of IgE^+^ and IgG1^+^ PCs. This result is consistent with studies that rapamycin could cause a reduction in serum IgE and IgG1 in mice induced by allergens (63–65). The calcium signaling pathway is another downstream of BCR. The basal intracellular calcium concentration in IgE^+^ PCs and IgE^+^ GC-like B cells elevates due to the autonomous activation of Syk/BLNK (55, 65). Enhancing the calcium signaling in IgE^+^ PCs caused mitochondrial apoptosis mediated by BIM (65). Calcineurin-NFAT is downstream of calcium signaling and may contribute to IgE^+^ PCs differentiation rather than apoptosis (65) (Figure 2).

Both ectodomain and cytoplastic tail contribute to IgE-BCR signaling. Chimeric BCR containing transmembrane cytoplastic tail of IgG1 and ectodomains of IgE could induce PC differentiation and apoptosis; however, the ectodomains of IgG1 and transmembrane cytoplastic tail of IgE could not, implying that the ectodomains of IgE-BCR contributed to downstream signaling spontaneous activation (55). Moreover, EMPD of IgE-BCR could interact and activate CD19, thus promoting PC differentiation. As mIgE_S_ isoform lacking EMPD could also activate downstream signaling of BCR without stimulation, there should be other alternative regions for spontaneous activation. The domains CH1-CH4 of IgE are sufficient to active Syk and BLNK in chimeric BCR without EMPD (55). More recently, the ITT motif of IgE-BCR has been demonstrated to promote IgE surface expression, accumulation of IgE^+^ PCs, and memory IgE responses in mice (60). As mentioned, the ITT motif could enhance calcium signaling and Erk MAP kinase pathways. Several studies found that Erk MAP kinase pathways did not play a significant role in IgE^+^ PCs differentiation and apoptosis (55, 65). ITT motif may contribute to IgE^+^ PCs maintenance through calcium signaling pathways. In addition, ITT promotes IgE surface expression, which may facilitate the activation of IgE-BCR (60).

The molecular mechanisms based on mouse models or mouse cell lines are associated with mIgE_S_. To clarify the distinct functions of mIgE_L_ and mIgE_S_, Vanshylla et al. (56) generated mIgE_L_ and mIgE_S_ expressing cells. They found that mIgE_L_-expressing cells had much weaker signaling of PI3K/Akt, MAPK, and calcium signaling pathways. Moreover, compared with full-length IgE-BCR, IgE-BCR with EMPD deletion significantly promotes murine B cell apoptosis (58). As discussed above, autonomous activation of mIgE_S_ signaling promotes SLPCs differentiation and apoptosis; impaired mIgE_S_ function contributes to LLPC generation and long-lasting serum IgE titer (55). Whether weaker signaling mediated by mIgE_L_ could compete and attenuate the mIgE_S_ pro-apoptosis effect remained to be elucidated. If that is the case, the ratio mIgE_L_/mIgE_S_ on PCs surface may be implicated in the survival of PCs.

## Discussion

Clinical observations indicate the responsibility of IgE^+^ LLPCs for persistent allergies, but the characteristics of IgE^+^ LLPCs are poorly studied, especially in humans. IgE^+^ LLPCs could be induced in the allergic mouse model, but only indirect evidence supports the presence of IgE^+^ LLPCs in allergic patients. IgE^+^ LLPCs mainly reside in BM and are lesser in the spleen and mucous. The prosurvival factors in the BM have been extensively studied, but a few focus on the mucosal microenvironment where IgE^+^ LLPCs exist. In non-atopy conditions, IgE^+^ B cells showed the propensity to differentiate into SLPCs due to the autonomous activation of IgE-BCR signaling. It is interesting to investigate the change of these signaling pathways in subjects with persistent allergies. In addition, mIgE_L_ and mIgE_S_ deliver altered signaling, which may play distinct roles in IgE^+^ PCs survival. Applying mutant mice expressing mIgE_L_ isoform helps to explore the unique role of mIgE_L_ in allergic conditions.
